# Genetic Determination of the Amount of White Spotting: A Case Study in Siberian Cats

**DOI:** 10.3390/genes13061006

**Published:** 2022-06-02

**Authors:** Agnieszka Górska, Wioleta Drobik-Czwarno, Agata Górska, Joanna Bryś

**Affiliations:** 1 Department of Chemistry, Institute of Food Sciences, Warsaw University of Life Sciences, Nowoursynowska St. 166, 02-787 Warsaw, Poland; agata_gorska@sggw.edu.pl (A.G.); joanna_brys@sggw.edu.pl (J.B.); 2 Department of Animal Genetics and Conservation, Institute of Animal Sciences, Warsaw University of Life Sciences, Nowoursynowska St. 166, 02-787 Warsaw, Poland; wioleta_drobik@sggw.edu.pl

**Keywords:** white spotting, cat, coat color, inheritance, pedigrees

## Abstract

The current hypothesis, along with the opinion of the breeders, is that a cat with two copies of the white spotting allele (*SS*) has white on more than half of its body, while a cat with only one copy (*Ss*) has white on less than half of its body. The present study was based on the analysis of two large pedigree databases of Siberian cats (23,905 individuals in PawPeds and 21,650 individuals in Felis Polonia database). The distribution of the amount of white spotting in the offspring of cats with different amounts of white was investigated. Significant differences compared to expected distributions were observed. In many cases the amount of white in cats that were supposed to be homozygous was less than 50% of the body, while in many supposedly heterozygous cats a very large amount of white (over 50%) was observed. This phenomenon was also presented on the verified examples of the specific families excluding possible errors in determining the amount of white by the breeder. The collected evidence suggests that there are other factors involved in the inheritance of the amount of white in cats and the current hypothesis should be revised.

## 1. Introduction

Color variations of cat coat have been one of the main interests of the breeders and cat owners for decades. Scientists started to investigate the inheritance of cat colors in the early twentieth century [1]. As a result, large number of genetic tests is currently available for the domestic cat, such as Siamese colorpoint, dilute coat color or agouti [2,3]. Despite that, knowledge about the inheritance of the amount of white spotting is still very limited. 

White spots are present in many mammals such as horses, mice, cattle, dogs etc. [4,5,6,7,8]. In 1919, Whiting [9] mated cats and studied the inheritance of varying amounts of white-spotting. His work suggested that there is an allelic series of solid-white, much spotted, little spotted and full solid color, with dominance in the degree of decreasing pigmentation. Molecular research based on microsatellite markers has shown that a white phenotype in cats, like in many other species, is associated with mutations in the *KIT* gene [10]. David et al. [11] determined that two different retroviral insertions in intron 1 of the *KIT* gene are responsible for white and white-spotted phenotypes in cats. Full length feline endogenous retrovirus (*FERV1*) element is associated with white spotting, while *FERV1* long terminal repeat is associated with all dominant white individuals. This was confirmed later on by an independent investigation and whole genome sequencing [12]. As was proposed by David et al. [11] three alleles are responsible for white phenotype at W locus: Dominant White (*W*), white spotting (*w^s^*) and wild-type (*w+*). The degree of white pigmentation appears to be associated with homozygosity vs heterozygosity for *w^s^* allele.

**Figure 1:**
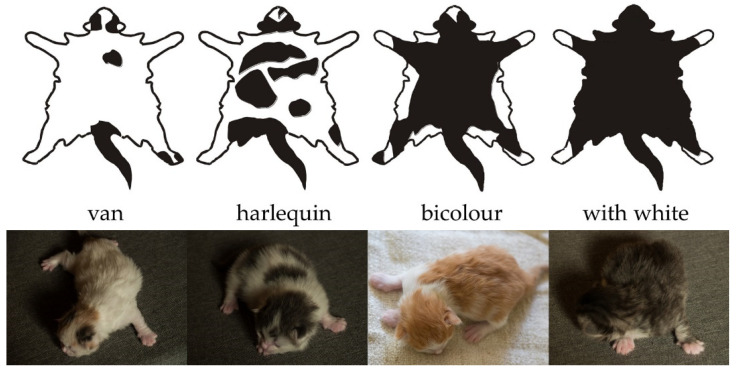
Examples of the distribution of white spotting in van (EMS code 01), harlequin (02), bicolor (03) and unspecified amount of white (09) according to Fédération Internationale Féline d’Europe [13].

Felinology organizations divide the amount of white spotting in cats into a few patterns; usually these are van, harlequin, bicolor and unspecified amount of white (Figure 1) [13,14,15]. This division assumes that van (EMS code 01) has white on approximately more than 75% of body, harlequin (02) between 50–75%, bicolor (03) 25–50% and cats with an unspecified amount of white (09) less than 25%. Other amounts like mitted (04) and Snowshoe (05) pattern are recognized in Birman, Ragdolls and Snowshoe breeds [13,14,15]. A separate recessive allele (*G*), situated also in the *KIT* gene, has been identified for Birman white gloving [16].

Currently, breeders along with felinology organizations adopted an inheritance model according to which full solid color (*ss*) is recessive. Heterozygote *Ss* is postulated to possess restricted areas of white spotting (less of the body): usually the feet, nose, chest, and belly, while the *SS* homozygote is supposed to have white regions covering most of the body [17,18,19,20]. This s and *S* alleles were introduced by Cooper et al. [10] and correspond to *w^+^* (*s*) and *w^s^* (*S*) alelles postulated be David et al. [11] who identified actual genetic variants. Dominant white is caused by a different retrovirus insertion in *KIT* gene [11] and feline organizations usually treat this trait as if it is controlled by a different locus with two alleles, W and w. Therefore, genotypes of cats with full solid color (without white) according to different classifications can be listed as *w+w+, ww* or *ss*.

The information about the different degrees of white pigmentation in siblings that inherited the identical *w^s^* allele can be found in the literature, and the influence of other genetic modifiers on white-spotting expression has been suggested [11]. Therefore, although a number of segregation studies and sequence analysis were performed over the years, the inheritance mechanisms of the amount of white spotting is still not fully understood. Additionally, this problem has not been described so far for large pedigreed populations. To address this issue, our study tested whether inheritance of white spotting in Siberian cats deviates from Mendelian inheritance.

## 2. Materials and Methods

### 2.1. Data Collection

Siberian Cat breed was selected for the analysis as they are characterized by the highest genetic diversity among the breeds [21] and big variation in the amount of white spotting. In this breed, any amount of white is present and accepted by felinological organizations [13,14,15]. The amount of white spotting has a gradually changed character. The official categorization used by felinology organizations was used in our analysis (van, harlequin, bicolor, with white and without white) as this is the only available data. All pedigree data regarding Siberian cats were obtained from PawPeds [22] and Felis Polonia [23] databases. PawPeds is an international organization that provides pedigree databases for many breeds. The Siberian cat database contains pedigrees of cats from all over the world. It is focused mainly on breeding cats and contains only a few data records on whole litters. Felis Polonia is a Polish organization affiliated to the Fédération Internationale Féline d’Europe. It contains data from the largest number of catteries in Poland and has the most complete pedigrees of Siberian cats. It is worth mentioning that whole litters are included in this database.

The collection of data was focused on the amount of white spotting. Available database contained information on 23,905 individuals in PawPeds and 21,650 individuals in Felis Polonia database (Supplementary FPL and PawPeds database). The number of cats used in matings analysis was equal to 13,244 in PawPeds and 12,279 in FPL (INFO field in Supplementary Materials). The correctness of the pedigrees has been checked. Individuals without a complete data set or with incorrect data were excluded from the analysis of offspring distributions. Pedigrees with both documented parents were considered complete. In the case of some cats, especially from the time of the beginning of the breed, there was no information about the parents or about the parents’ colors in their pedigrees. Cats from the novice class did not have parents in their pedigrees as they were new individuals introduced into the genetic pool. Cats from the novice class can be used in the breeding program after fulfilling the feline organization requirements (have to obtain the qualification “Excellent” for recognized breed according to the target breed standard from the judges at international show). The novice class for Siberian cats can be only accepted for cats born in the former Union of Soviet Socialist Republics (USSR).

In spite of excluding cats with incorrect pedigrees, some inexperienced breeders may have incorrectly determined the amount of white spotting, especially in colorpoint cats. The colorpoint gene [24] is present in the Siberian breed. This can cause problems with determining the amount of white in the first weeks of life in red and cream kittens. The colorpoint mutation is temperature-sensitive and produces pigment only at the cooler extremities of the body, causing a “mask” on the face and darkened paws and tail. Kittens are born all white; in the first days of life the color begins to appear in kittens with black pigment, which makes it possible to determine the amount of white. In kittens with red pigment, this is almost impossible, as they often remain very bright until they are few months old. It is only possible to determine whether they are with white, but not to determine the amount of white. Results without red and cream colorpoints are shown in Supplementary Tables S1 and S2.

We used an in-house script to found pedigrees in which the colors of the offspring did not correspond to the rules of inheritance. Pedigrees with valid inheritance rules were considered correct. Completely white cats were also excluded from the analysis because it is not possible to identify if and how many white spottings are present on their bodies. Only data of litters with complete and correct information about parents and offspring were included.

Genotypes were assigned as *SS*, *Ss* or *ss* according to the current hypothesis about the inheritance of white spotting based on the phenotypic classification to van (*SS*), harlequin (*SS*), bicolor (*Ss* or possible in some cases *SS*), with white (*Ss*) and without white (*ss*).

### 2.2. Statistical Analysis

Distribution of white spotting in offspring was shown for selected matings. The chi-square and binomial tests for proportion were used for statistical confirmation of segregation ratios according to Mendelian inheritance. A *p*-value < 0.05 was considered statistically significant.

## 3. Results

To verify whether the principles of Mendelian inheritance of the coat color could be applied, we used a number of crosses between males and females with different amounts of white (Table 1 and Table 2).

The low number of 01 and 02 cats was taken into account due to their low representation in the population. The highest percentage of 01 and 02 was observed in 02 x 09 matings for both datasets. Bicolor (03) cats were the most common progeny in 03 x 03 matings, while cats with white (09) were observed in 09 x 09 (Table 1) or 09 x ww (Table 2).

Chi square test was performed for the probability distribution in the offspring (Table 3). Due to inconsistencies in the classification of bicolor cats–below or above 50% of white–binomial test for proportion was performed only for the expected proportion of without white cats for each mating. The large number of differences between observed and expected proportion of cats representing each phenotype was determined and confirmed with highly significant test results (Table 3).

Results excluding red and cream colorpoints did not differ from those obtained including red and cream colorpoints (Supplementary Tables S1 and S2).

## 4. Discussion

Homozygosity vs. heterozygosity for the *S* allele appears to have substantial influence on the degree of white pigmentation. According to our current knowledge, the amount of white is determined by the mutation in the *KIT* gene with two alleles, *S* and *s* and the following genotypes *SS, Ss, ss*. Homozygotes with two copies of *S* allele are expected to have white on most of the body, heterozygotes *Ss* have less on the body, while cats without *S* allele (*ss*) are full color. This hypothesis is in accordance with the common opinion within the breeding society, that is, the cats with a large proportion of white must have both parents with white. Some breeders, however, don’t agree with this because a high number of matings results in phenotypes of the offspring that are different from what is expected.

According to the hypothesis that a homozygous cat has white spotting on more than half of the body, and a heterozygous less than half, 25% of cats with a lot of white (01 and 02) is expected when mating two cats with white (09 x 09). In the performed analysis of the pedigree databases, it was observed that the number of such cats was 1.3% for PawPeds and 0.7% for FPL, respectively (Table 3). This suggests that the hypothesis used so far does not match the actual amount of white spotting in the offspring. Additionally, we should get 25% of kittens without white in this combination, which is confirmed by the PawPeds database, but in the FPL database there was a significant difference in the number of cats without white.

The same distribution of the progeny should be obtained by combining two bicolor cats (03 x 03), but the result of the analysis also significantly differs from the assumptions. The distribution of cats with different amounts of white and without white differs from the distribution for the mating of two cats with white (09 x 09), which may be due to the small number of offspring available from such mating. Additionally, it may also result from imprecise determination of the amount of white by breeders, especially in cats with amount of white close to 50% (which is the border between proposed genotypes *Ss* and *SS*). Occasionally, more conservative breeders attribute color 03 to cats that have more than 50% white on their bodies. Some organizations also allow the use of the term bicolor for cats with an amount of white up to 60%, for which an *SS* genotype should be assumed. It means that 03 phenotype is not fully reliable. With more than 50% white spotting, breeders are very careful and only assign harlequin to cats when there are clearly separated patches of color on the body. Therefore, we consider the color 01, 02, 09 and without white to be most reliable.

In the case of mating a harlequin cat with a cat with white (02 x 09), we would expect 100% offspring with white, including 50% with the *Ss* genotype and 50% with *SS*. The data, however, revealed significant differences in the offspring distribution, including the appearance of a large number of cats without white (6.0% for PawPeds and 18.5% for FPL). This suggests that some of the harlequins are heterozygous, which is also not in line with the current hypothesis.

Another piece of evidence for the different scheme of inheritance of the amount of white spotting is the appearance of cats with large amounts of white, such as vans and harlequins among the offspring of cats with less white and cats without white (09 x ww). In general, the mating of cats without white gives an interesting perspective. The offspring of the harlequin cat and the cat without white should give 100% white spotting cats, with the amount of white up to half the body. The result of the analysis showed that there were only 76.2% of such cats in the PawPeds database and 49.1% in the FPL, while a large number of cats without white was observed. Additionally, a large number of cats with more white spotting can be seen in the offspring.

These results suggest that there are other factors involved that can affect the inheritance of the amount of white. Novel mutations within the *KIT* gene are still being discovered in other species such as cattle [4] or horses [6] and it’s possible that additional changes in a cat’s *KIT* gene are yet to be found. Other genes and interactions between them may be also involved [8,9,10,11]. The distribution of white spotting is determined primarily by melanoblasts development and migration and therefore other complex gene pathways that are determining early development may be involved. A number of genes have been associated with the amount of white spotting over the years, such as *EDNRB, MITF* or *TRPM1*. It is possible also that different mechanism of inheritance can determine coat colour such as epigenetic changes involving imprinting [8].

**Figure 2:**
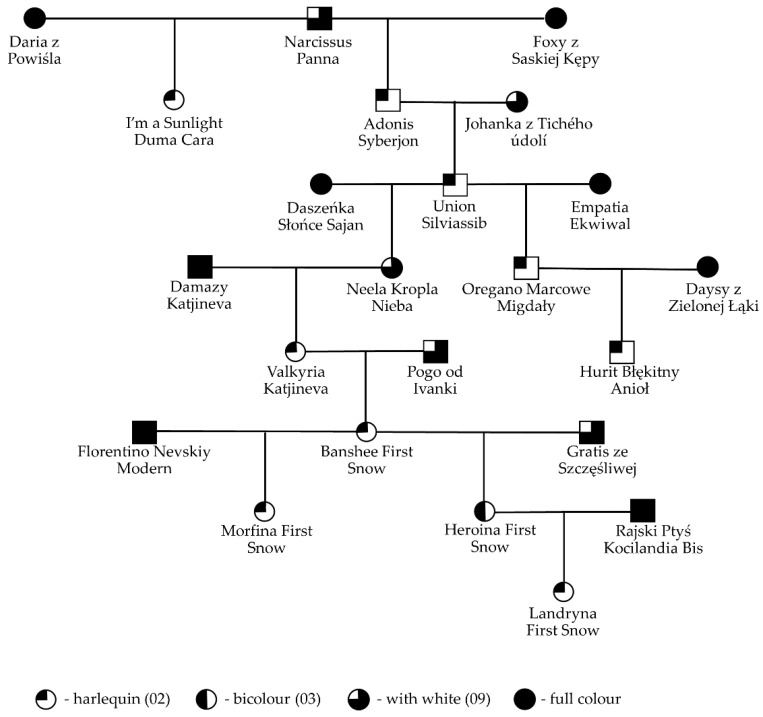
Representative pedigree for large amounts of white spotting inheritance. All cats with white spotting gene (including Union Silviassib, Banshee First Snow and Heroina First Snow) are heterozygous cats (*Ss*) based on phenotype and pedigree information.

**Figure 3:**
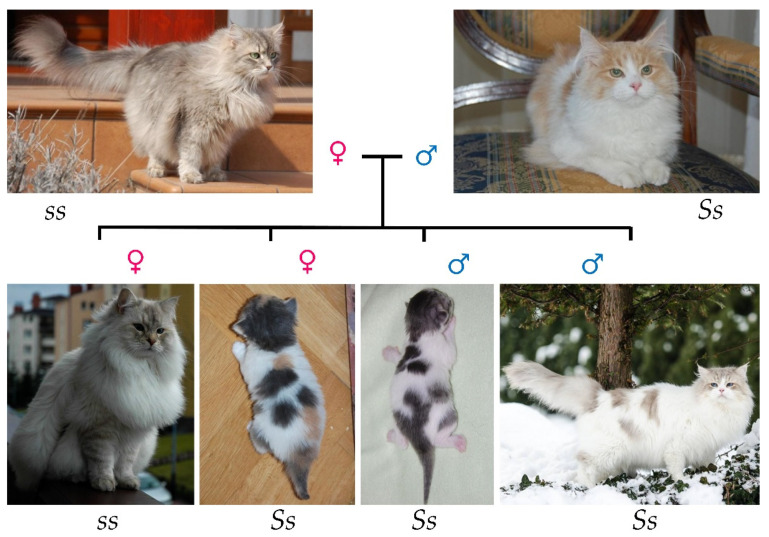
Pictures of progeny of heterozygous (*Ss*) harlequin male Union Silviasib and full color (*ss*) female Empatia Ekwiwal. Genotypes were determined based on phenotype and pedigree information. The mating resulted in three harlequins kittens and one full color kitten. The photos of Neva Masquerade (colorpoint variation of siberian cat) kittens are taken of adult cats, because the exact distribution of color is better seen when they are adult [24]. The chest of the first offspring from left seems to be almost white. It is an impression due to the colorpoint coat. This is typical of these color pattern, especially when the cat has a winter coat [13,14,15,24].

Apart from the offspring distributions, many representative pedigrees with specific examples that deny the current hypothesis have been prepared. Figure 2 shows a representative multigenerational pedigree of cats with a large amount of white spotting inherited, in many cases, from matings with cats without white. All cats in the pedigree shown are heterozygous; either they had a parent without white or their offspring was without white (the pedigree does not include all offspring). Narcissus Panna, a male with white, gave 02 kittens with multiple females without white. One of these examples is his son Adonis Syberjon (02, *Ss*) which possessed offspring that were both harlequin and without white. This tendency continued in the following generations: his son Union Siviassib (02, *Ss*) had offspring that were both harlequin and without white (Figure 3), the same as his son Oregano Marcowe Migdały (02, *Ss*). Neela Kropla Nieba, daugther of Union, was only with white (09), but she possessed harlequin and without white offspring. The next generations (Valkyria, Banshee and Heroina) were harlequin (02, *Ss*). Banshee and Heroina gave both kittens with a high amount of white (02) and kittens without white. Figure 3 shows pictures of the whole litter, whose parents are Union Silviasib and Empatia Ekwiwal (see Figure 2). Three kittens are harlequins and one kitten is without white. One of the kittens (Oregano Marcowe Migdały, first picture from the right in Figure 3) became a breeding male and gave many harlequin kittens (02, *Ss*) with females without white.

Many crosses that don’t fit the standard inheritance pattern have been observed over the generations. For example, van male Meldgaards Nikolaz (01) gave harlequin (02, *Ss*) offspring when he mated with two different females, both without white. One of the offspring was Lubov Sibiri Sergeij Nikolaevich (02, *Ss*) which gave offspring without white when he mated with without white female Majuns Luna. This also confirms that he was a heterozygote. What’s interesting is that one of his without white daughters was mated with bicolor male Zimas Thyson (03) and gave van male, Topmix Maximilian (01). This is not an isolated case.

Other examples are Nikopeja’s ManekiNeko Mi-Ke (02, *Ss*): her parents were with little white (09) and full color. Russian Irbis Svarog (01) gave two van kittens (01, *Ss*-Nenets Land Olivia and Nenets Land Noa) with two different females without white. Sjakkmatt’s Bowser (09, *Ss*) was mated with a female without white and gave van kitten Klockarbackens Bill (01, *Ss*). Sweet Honey’s Enya (03, *Ss*) and Milashka’s Gedeon (*ss*) gave van kitten Nackby’s Timbuktu (01). Jazz Wild Taiga (02, *Ss*) had a harlequin (02, *Ss*) kitten with female without white, and also had multiple van progeny with females with white. In subsequent generations for Jazz Wild Taiga, a lot of harlequin kittens (02, *Ss*) appeared in mating with cats without white. 

Such phenomena as those mentioned above can also be found in other breeds in which every variation in the amount of white spotting is present. Examples of such matings can be easily found, e.g., in available online in the PawPeds pedigree databases [25].

## 5. Conclusions

White spotting inheritance has been investigated in the last century, but it has not been fully understood. It is assumed that cats with a large amount of white have both parents with white, which would be the case with simple Mendelian inheritance. As it was shown in the present work, many exceptions occur within such matings. Additionally, specific pedigree examples show that the amount of white is inherited with additional factors that can stay hidden through the generations. Our results suggest that this phenomenon needs more attention to reveal the inheritance mechanism and help breeders in their breeding plans. Further molecular research is needed to identify other mutations or genes that are responsible in the determination of white spotting in cats.

## Figures and Tables

**Table 1:** Distribution of the offspring from selected crosses in FPL database according to proposed genotypes. Number of 01 x ww crossings unavailable (*N* = 12,279).

	*SS*		*Ss*		*Ss*		*ss*		
Parents	01 and 02		03		09		ww		Total
	*N*	%	*N*	%	*N*	%	*N*	%	
09 x 09	17	0.7%	137	5.9%	1312	56.6%	854	36.8%	2320
03 x 03	15	9.6%	77	49.4%	37	23.7%	27	17.3%	156
02 x 09	22	27.2%	14	17.3%	30	37.0%	15	18.5%	81
02 x ww	21	13.2%	37	23.3%	41	25.8%	60	37.7%	159
03 x ww	24	1.6%	414	28.1%	481	32.6%	556	37.7%	1475
09 x ww	10	0.1%	142	1.8%	3450	42.7%	4486	55.5%	8088

*SS*–homozygote, *Ss*–heterozygote, *ss*–without white, 01–van, 02–harlequin, 03–bicolor, 09–with white, ww–without white.

**Table 2:** Distribution of the offspring from selected crosses in PawPeds database according to proposed genotypes (*N* = 13,244).

	*SS*		*Ss*		*Ss*		*ss*		
Parents	01 and 02		03		09		ww		Total
	*N*	%	*N*	%	*N*	%	*N*	%	
09 x 09	31	1.3%	199	8.3%	1573	65.8%	589	24.6%	2392
03 x 03	42	15.4%	121	44.5%	60	22.1%	49	18.0%	272
02 x 09	30	20.1%	41	27.5%	69	46.3%	9	6.0%	149
(01 or 02) x ww	39	11.6%	94	28.1%	161	48.1%	41	12.2%	335
03 x ww	49	2.4%	544	26.6%	642	31.4%	810	39.6%	2045
09 x ww	20	0.2%	294	3.7%	3806	47.3%	3931	48.8%	8051

**Table 3:** Statistical tests for independence between given and theoretical distributions according to Mendel’s law.

		*SS*		*Ss*		*ss*			
Parents	Dataset	01 and 02		03 or 09		ww		Chi-Square	Binomial
		Expected	Observed	Expected	Observed	Expected	Observed	*p*-Value	*p*-Value
09 x 09	FPL	25%	0.7%	50%	62.5%	25%	36.8%	<0.001	<0.001
	PawPeds		1.3%		73.8%		24.6%	<0.001	0.688
03 x 03	FPL	25%	9.6%	50%	73.1%	25%	17.3%	<0.001	0.026
	PawPeds		15.4%		66.6%		18.0%	<0.001	0.008
02 x 09	FPL	50%	27.2%	50%	54.3%	0%	18.5%	<0.001 ^i^	<0.001
	PawPeds		20.1%		73.8%		6.0%	<0.001 ^i^	<0.001
02 x ww	FPL	0%	13.2%	100%	49.1%	0%	37.7%	-	<0.001
	PawPeds		11.6%		76.2%		12.2%	-	<0.001
03 x ww	FPL	0%	1.6%	50%	60.7%	50%	37.7%	<0.001	<0.001
	PawPeds		2.4%		57.6%		39.6%	<0.001	<0.001
09 x ww	FPL	0%	0.1%	50%	44.5%	50%	55.5%	<0.001	<0.001
	PawPeds		0.2%		51.0%		48.8%	0.036	0.059

^i^–test performed for proportion of *SS* vs. *Ss* or *Ss* vs. *ss* accordingly.

## Data Availability

Data available in a publicly accessible repository at: https://www.pawpeds.com/db/?p=sib accessed on 30 September 2021 and https://ssl.felispolonia.eu/index.php?r=9 accessed on 30 September 2021.

## Supplementary Materials

The following supporting information can be downloaded at: https://www.mdpi.com/article/10.3390/genes13061006/s1, Table S1: Distribution of the offspring from selected crosses in FPL database according to proposed geneotypes. Red and cream colorpoints are excluded from this anlysis. Number of 01 x ww crossings unavailable; Table S2: Distribution of the offspring from selected crosses in PawPeds database according to proposed geneotypes. Red and cream colorpoints are excluded from this anlysis; FPL Database, PawPeds Database.
